# Delayed decisions—how long is too long?

**DOI:** 10.1093/jhps/hnw029

**Published:** 2016-08-17

**Authors:** Richard Villar

**Affiliations:** Journal of Hip Preservation Surgery*Correspondence to: R. Villar. E-mail:jhps.editorialoffice@oup.com

Despite being in publishing for the best part of two decades there is one thing I cannot adequately explain. Why is it that when a journal wants something from an author, the journal gives a deadline of barely a week but when an author wants something from a journal he can seemingly wait for ever? A typical example is when waiting for review. An author spends a year, or more, undertaking a research project, he writes it up, dots every ‘i’ and crosses every ‘t’, takes a deep breath, crosses his fingers and then sends it in. The journal may send out an acknowledgement of receipt but then the wait begins. Therein lies the problem; the review time.

In approximately 1980 the median time between submission and acceptance was of the order of 70 days for the 4353 journals that made up the PubMed database at that time. By 2015, this delay had risen to 100 days for the then 9045 journals on PubMed. This delay has hovered around the 100-day mark for almost a decade.^1^ However, this is a median figure. Some journals can boast a delay between submission and acceptance of as little as 7 days, others hang their heads and confess to a delay of over 130 days.^2^ There is some evidence to suggest that journals with very high and very low impact factors have longer delays between submission and acceptance. Meanwhile production time has decreased, most likely because of improvements in technology.

There are several issues that lead to a delay in a decision being made about an article. In many respects at *JHPS* we are fortunate as our reviewers—each utterly brilliant in my view—are specialists in the world of hip preservation. I note how so many other journals, some highly established, struggle when seeking a reviewer for an article concerning hip preservation. At *JHPS* we are so very lucky. We have many reviewers, all voluntary, all invaluable, and requests come in regularly to be added to the journal’s reviewer database. Never once have we had to ask an author to recommend a reviewer. To do so worries me anyway, as one of the latest scams in the world of scientific publishing is for an author to recommend a reviewer but actually to fabricate the recommendation, supplying a false email address so the author ends up by reviewing their own article and recommending acceptance. I am not joking. At *JHPS* we see none of that, nor would I expect to do so.

So how long is too long? That is impossible to say. However, if a recent internal debate among members of the World Association of Medical Editors is anything to go by, one journal held onto a submission for 5 months before sending it out for review. To me that is inexcusable. The general feeling was that if a journal takes longer than 3–6 months to make a decision, then you should be looking elsewhere to publish.

But specialist reviewers are busy people and they do the job for love. It takes time to review a submission, in some cases the best part of half a day. How many of us have that amount of time to spare in an otherwise frenetic existence? Not many I would suggest, which makes our reviewers remarkable people. Surprisingly, there is a lack of association between the quality of a review—generally first class for *JHPS*—and reviewer characteristics. For example, one might imagine that individuals actively involved in research, academic orthopaedics, or members of research-funding bodies would make better reviewers. This does not appear to be the case, a feature that was well noted in 1998 by Black *et al.*^3^

Once reviewed, very few articles are accepted first time. It is totally normal for an article to be sent back to the authors for revision once, twice, sometimes thrice and, on occasion, even more. After every revision comes another review, ideally by the same reviewers who saw the submission on the first occasion, but not always so. And full agreement? No way. That is most unusual. The editor treads that dangerous No-Man’s Land between reviewers’ opinions, trying hard to make sense of expert decisions and to reach a fair judgement. We do our best, although cannot always pretend to get it right. But the multiple submissions, the toing and froing of behind-the-scenes editor/reviewer debate adds time. Meanwhile the author is back at base, perhaps not understanding why there is such delay when, in fact, their article is the topic of heated discussion among his peers. This is one of the problems of blinded review, where journals act in a similar way to Secret Services and authors tread gently in order to avoid upsetting the eventual decision. As Editor-in-Chief, please let me reassure you. Do chase the journal if you feel we are taking too long—we are not perfect—and have no fear whatsoever that your query will in any way influence the eventual decision. It will not.

Turning to the last issue of *JHPS* (issue 3.2), again we were blessed by the usual collection of brilliance, from which I find it repeatedly impossible to choose the best. However, of real interest to my own practice was the article from Wilson and Keene^4^ on the treatment of ischiofemoral impingement, a diagnosis that for some reason is becoming more frequent globally, perhaps because we now recognise the existence of this mysterious entity we abbreviate to IFI. It appears I must no longer rely on injection to resolve the problem and should think more about an arthroscopic iliopsoas tenotomy in combination with resection of the lesser trochanter. The other article that specifically attracted my attention was the report from the Danish Hip Arthroscopy Registry.^5^ It makes a strong case for the development of such a registry and is a lesson to us all. Very well done to Mygind-Klavsen *et al.* who put the article together and are doing much to show us the way with registries. For those other registries in the world that I realise also exist, indeed to those that are just starting out, do please send us your work and data. We would love to look at it.

And for this issue, number 3.3? Again, we are spoilt for choice. For some reason I have been seeing numerous cases of avascular necrosis (AVN) in the past year, perhaps because of increasing periods spent outside my shortly-to-become-non-European-Union country of the United Kingdom. Consequently the article by Vaishya *et al*.^6^ from New Delhi on the use of a sartorius muscle pedicle iliac bone graft is especially helpful. They report an up to 80% success. I was also fascinated by an article from The Netherlands, by Röling, Mathijssen and Bloem^7^ in which they establish that 0.44% of the patients seen by a general practitioner will present with groin pain. Of these, 17% will have femoroacetabular impingement. It is good to have an idea of how much work there is wandering the streets outside our various surgery doors.

And let me slip this one in as well, otherwise you might miss it. I beg of you to go to Ajay Malviya’s What the Papers Say section and look at his very last entry. I admit that the International Journal of Biometeorology is not normally at the top of my reading list. However, an article from Hungary^8^ has made a strong case for the use of a sulphur bath (balneotherapy) in the management of osteoarthritis of the hip. Perhaps our osteoarthritis patients should take to the waters immediately. It was a randomised controlled trial to boot.

My very best wishes to you all.
